# Orchestrated Engineered Polyphenol‐Peptide Condensates Coupled With β‐GP Reverses Diabetic Osteoporosis by Remodeling Mitophagy

**DOI:** 10.1002/advs.75666

**Published:** 2026-05-12

**Authors:** Xiuyun Xu, Meiqin Zhang, Ting Wu, Erfan Wei, Andrei Y. Hancharou, Zeying Wang, Zijuan Wang, Letian Lv, Likun Wu, Xingtong Pan, Qiyue Zhu, Xinyi Dong, Hao Liu, Yongsheng Zhou

**Affiliations:** ^1^ Department of Prosthodontics Peking University School of Stomatology Beijing China; ^2^ The Central Laboratory Peking University School and Hospital of Stomatology Beijing China; ^3^ National Center of Stomatology National Clinical Research Center for Oral Diseases National Engineering Research Center of Oral Biomaterials and Digital Medical Devices Beijing Key Laboratory for Intelligent Biomanufacturing and Regeneration of Craniofacial Tissues Beijing Key Laboratory of Digital Stomatology NMPA Key Laboratory for Dental Materials Research Center of Engineering and Technology for Computerized Dentistry Ministry of Health Beijing China; ^4^ Institute of Biophysics and Cell Engineering National Academy of Sciences of Belarus Minsk Republic of Belarus

## Abstract

Diabetic osteoporosis (DOP) is a chronic complication of diabetes mellitus characterized by reduced bone mass, disrupted microarchitecture, and an elevated fracture risk. Persistent oxidative stress and inflammation further inhibit osteogenesis and angiogenesis, accelerating bone degeneration. In this study, we used a peptide‐polyphenol conjugation strategy to develop a multifunctional colloidal nanoplatform (β‐GP@EGCG‐E7). The bone‐targeting peptide E7 was covalently conjugated with epigallocatechin‐3‐ gallate (EGCG) and subsequently loaded with β‐glycerophosphate (β‐GP). Sequentially, this nanoplatform integrates bone‐targeted delivery, improving mitochondrial quality by anti‐inflammation and antioxidation, as well as osteogenesis and angiogenesis within a single system. In detail, in a high glucose microenvironment, the nanoplatform selectively accumulates in bone lesions, scavenges excess intracellular reactive oxygen species (ROS) triggered by high glucose, clears damaged mitochondria by activating PINK1‐Parkin mediated mitophagy, and exerts anti‐inflammatory effects to rebalance the microenvironment of bone regeneration. Concurrently, sustained phosphate release supports mineralization, promotes osteogenic differentiation, enhances angiogenesis, and improves local microcirculation. In diabetic osteoporotic models, β‐GP@EGCG‐E7 significantly reduced oxidative stress, restored mitochondrial homeostasis, and promoted bone and vascular regeneration. This study provides a promising therapeutic strategy for DOP and highlights the potential of peptide‐polyphenol hybrid nanomaterials for regenerative applications.

## Graphical abstract

A multifunctional β‐GP@EGCG‐E7 nanoplatform is engineered by polyphenol–peptide condensation and phosphate loading. Bone‐targeted delivery, antioxidant and anti‐inflammatory activity, and mitophagy restoration are integrated in one system, enabling protection of osteoblasts and endothelial cells and promoting bone regeneration under diabetic osteoporosis conditions.

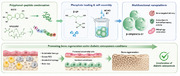

## 1 Introduction

Diabetes mellitus (DM) is a chronic hyperglycemic disorder caused by abnormal carbohydrate metabolism. It is frequently accompanied by complications affecting the eyes, heart, kidneys, nerves, and skeletal system [1, 2, 3, 4]. The global prevalence of DM has risen steadily, with an estimated 537 million individuals‐approximately 10% of the world's population‐affected in 2021, making it one of the most common chronic diseases worldwide [5, 6]. Among its complications, diabetic osteoporosis (DOP) has received increasing attention in recent years as a chronic musculoskeletal disorder. It is characterized by reduced bone mineral density (BMD), impaired bone microarchitecture, and an elevated risk of fragility fractures [7, 8]. The pathological mechanism of DOP extends beyond a simple imbalance in bone metabolism and is instead driven by a complex network sustained by persistent hyperglycemia. Specifically, hyperglycemia induces oxidative stress and inflammatory responses that suppress osteoblast differentiation and promote osteoclastogenesis, ultimately disrupting bone remodeling and leading to bone loss [9, 10]. Excessive glycation of bone collagen further impairs bone matrix mineralization by hindering mineral deposition and altering the matrix composition, thereby exacerbating skeletal fragility [11]. At the same time, endothelial injury induced by chronic hyperglycemia results in microcirculatory dysfunction, causing in insufficient perfusion, ischemia, hypoxia, and nutrient deprivation in bone tissue, all of which further accelerate the progression of osteoporosis [12]. Management of DOP remains challenging. Although certain antidiabetic agents effectively achieve glycemic control, some of them can also induce bone loss and increase fracture risk [13]. Conversely, several anti‐osteoporotic drugs may adversely effects on glucose metabolism [14]. These limitations highlight an urgent need for therapeutic strategies that concurrently address the metabolic disturbances of diabetes and the associated abnormalities in bone metabolism.

As multifunctional organelles, mitochondria are essential for cellular energy production, lipid catabolism, iron handling and heme synthesis, calcium signaling, and the regulation of both apoptosis and programmed necrosis [15]. The diabetic environment induces persistent mitochondrial damage, leading to the excessive generation of reactive oxygen species (ROS), which further disrupts cellular homeostasis [16, 17]. The functional integrity of mitophagy, the selective quality‐control process responsible for eliminating damaged mitochondria, is therefore crucial and pivotal in determining cellular fate [18, 19]. However, under diabetic conditions, mitophagy is frequently impaired, resulting in the accumulation of dysfunctional mitochondria, exacerbated oxidative stress, and further cellular injury. Among the regulatory mechanisms governing mitophagy, the PINK1/Parkin signaling pathway is recognized as a central mediator of mitochondrial quality control [20]. Recent studies have demonstrated the critical role of PINK1 in osteoblast differentiation [21, 22] and highlighted the therapeutic potential of enhancing osteoblastic mitophagy to counteract age‐related osteoporosis [23]. Consequently, targeted activation of mitophagy in the skeletal system has been proposed as a promising strategy for reversing diabetes‐induced bone damage.

Polyphenols are important nutritional components in the human diet. Among them, tea polyphenols have attracted substantial attention as functional ingredients because of their cost‐effectiveness, natural renewability, and favorable biosafety profile, and they exhibit notable anti‐inflammatory and antioxidant activities [24, 25]. Epigallocatechin‐3‐gallate (EGCG), the predominant polyphenol in green tea, possesses strong antibacterial and free‐radical‐scavenging properties. It has been shown to suppress the expression of pro‐inflammatory factors while increasing levels of the anti‐inflammatory cytokine interleukin‐4 (IL‐4) in lipopolysaccharide (LPS)‐induced macrophages in vitro [26, 27]. In recent years, research on tea polyphenols has expanded, shifting from their traditional use as standalone therapeutics to their emerging application as carriers in advanced drug delivery systems. The unique molecular structure of polyphenols confers exceptional supramolecular assembly capabilities, enabling stable interactions with biomolecules, particularly proteins and peptides, through non‐covalent forces such as hydrogen bonding, hydrophobic interactions, and π–π stacking, while preserving their biological activity [28]. This property provides an excellent foundation for the design and development of multifunctional biomaterials.

Recent advances in DOP‐ and osteoporosis‐directed biomaterials have demonstrated that nanomedicine can partially remodel the pathological bone microenvironment through several distinct strategies. For instance, tetrahedral framework nucleic acid (tFNA)‐based nanoparticles have been used to suppress ferroptosis and enhance the delivery of curcumin to bone marrow, thereby improving mitochondrial function and osteogenic differentiation in diabetic osteoporosis [4]. Gene‐activating framework nucleic acid systems have also been developed to upregulate SIRT1 and restore the osteoimmune microenvironment through macrophage polarization and NF‐κB inhibition [29]. More recently, targeted DOP nanoplatforms integrating glucose consumption, peroxide detoxification, and osteogenic ion release have further highlighted the therapeutic value of multifunctional design. However, most existing systems are still centered on one predominant intervention axis, such as ferroptosis suppression, osteoimmune regulation, glucose‐responsive local delivery, or ion‐mediated bone remodeling, and relatively few integrate mitochondrial quality control, bone‐specific accumulation, and sustained mineralization support within a self‐assembled therapeutic architecture.

**SCHEME 1:**
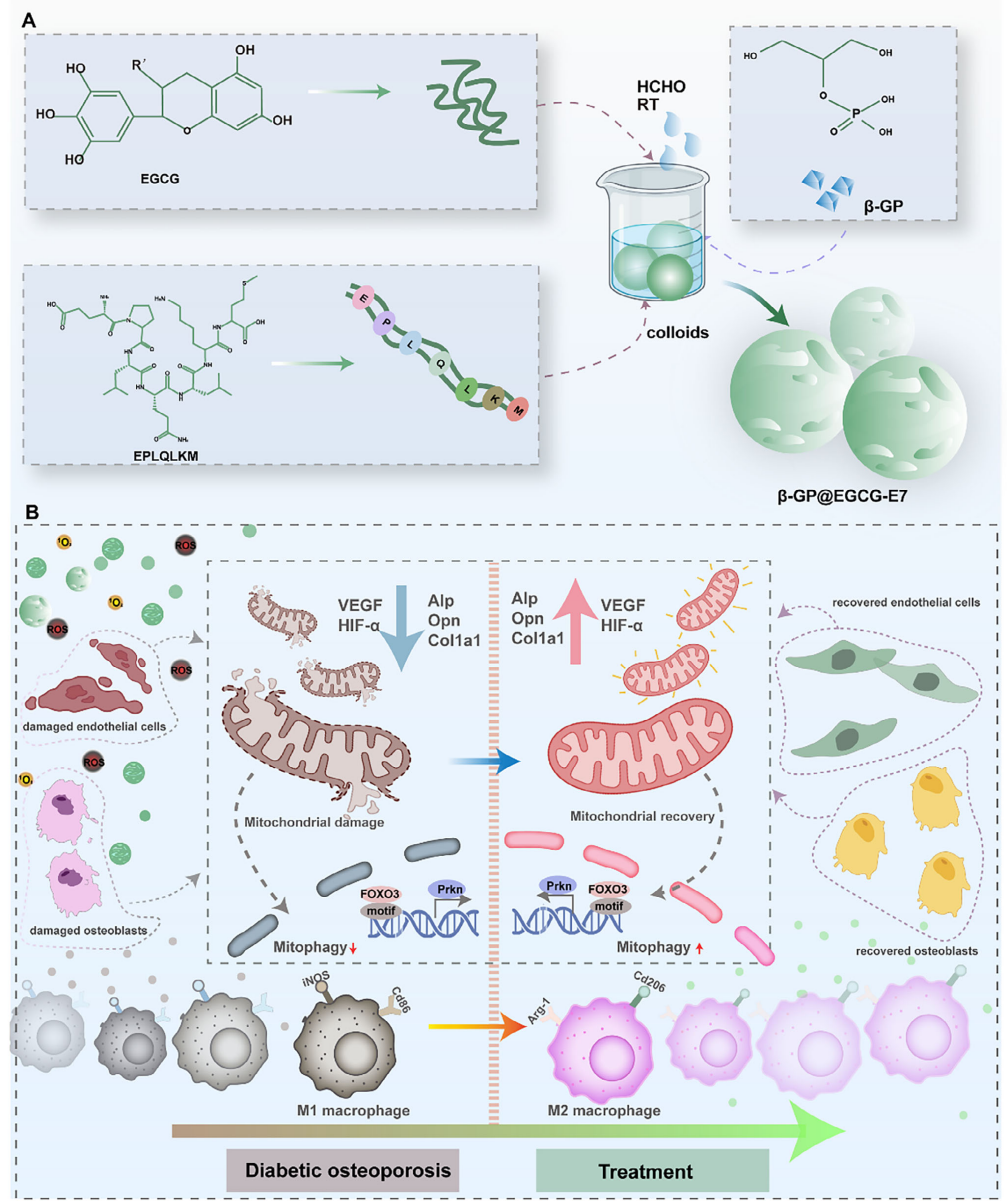
Preparation and therapeutic mechanism of β‐GP@EGCG‐E7 nanoparticles. (A) Schematic illustration of the preparation process for β‐GP@EGCG‐E7 nanoparticles. (B) Proposed therapeutic mechanism: β‐GP@EGCG‐E7 exerts antioxidant and anti‐inflammatory effects, restores mitophagy to alleviate mitochondrial damage in osteoblasts and endothelial cells, and thereby promotes bone tissue protection and regeneration.

Building on this foundation, we developed a supramolecular assembly system using EGCG in combination with a bone‐targeting peptide (E7). The E7 peptide enables the system to specifically recognize and home to bone tissue, leading to highly efficient localization within the bone marrow. This targeted delivery ensures that the therapeutic effects are concentrated at the desired site, thereby effectively alleviating mitochondrial dysfunction in bone cells and endothelial cells residing in the bone marrow microenvironment. To further address the complex pathological microenvironment of DOP, we constructed a fully biocompatible self‐assembled colloidal nanoplatform using a triple condensation strategy, in which the bone‐targeting peptide E7, natural EGCG, and the osteogenic precursor glycerophosphate (β‐GP) were co‐assembled into a unified nanostructure. Guided by the “structure‐as‐therapy” design concept, this system integrates molecular architecture with biological functionality, combining bone targeting, antioxidation and anti‐inflammation, angiogenesis, and osteogenic stimulation within a single platform. Mechanistically, the E7 peptide supplies specific affinity for bone tissue, enabling targeted accumulation and initiating a spatiotemporally ordered therapeutic cascade within the diabetic microenvironment. EGCG serves as a regulatory core by scavenging excess ROS and reactivating mitophagy through the PINK1‐Parkin signaling pathway, thereby alleviating oxidative stress and restoring cellular homeostasis. Subsequently, β‐GP undergoes enzymatic hydrolysis by alkaline phosphatase (ALP) to release phosphate ions continuously, promoting bone mineralization and osteoblastic differentiation and ultimately achieving a coordinated transition from metabolic recovery to structural regeneration. Overall, this self‐assembled nanoplatform embodies a structure‐function‐therapy integration paradigm, enabling synergistic regulation of redox balance and bone remodeling and providing a promising translational strategy for the precise and long‐term treatment of DOP (Scheme 1).

## 2 Results

### 2.1 β‐GP@EGCG‐E7 Nanocomplex Synthesis and Characterization

**FIGURE 1:**
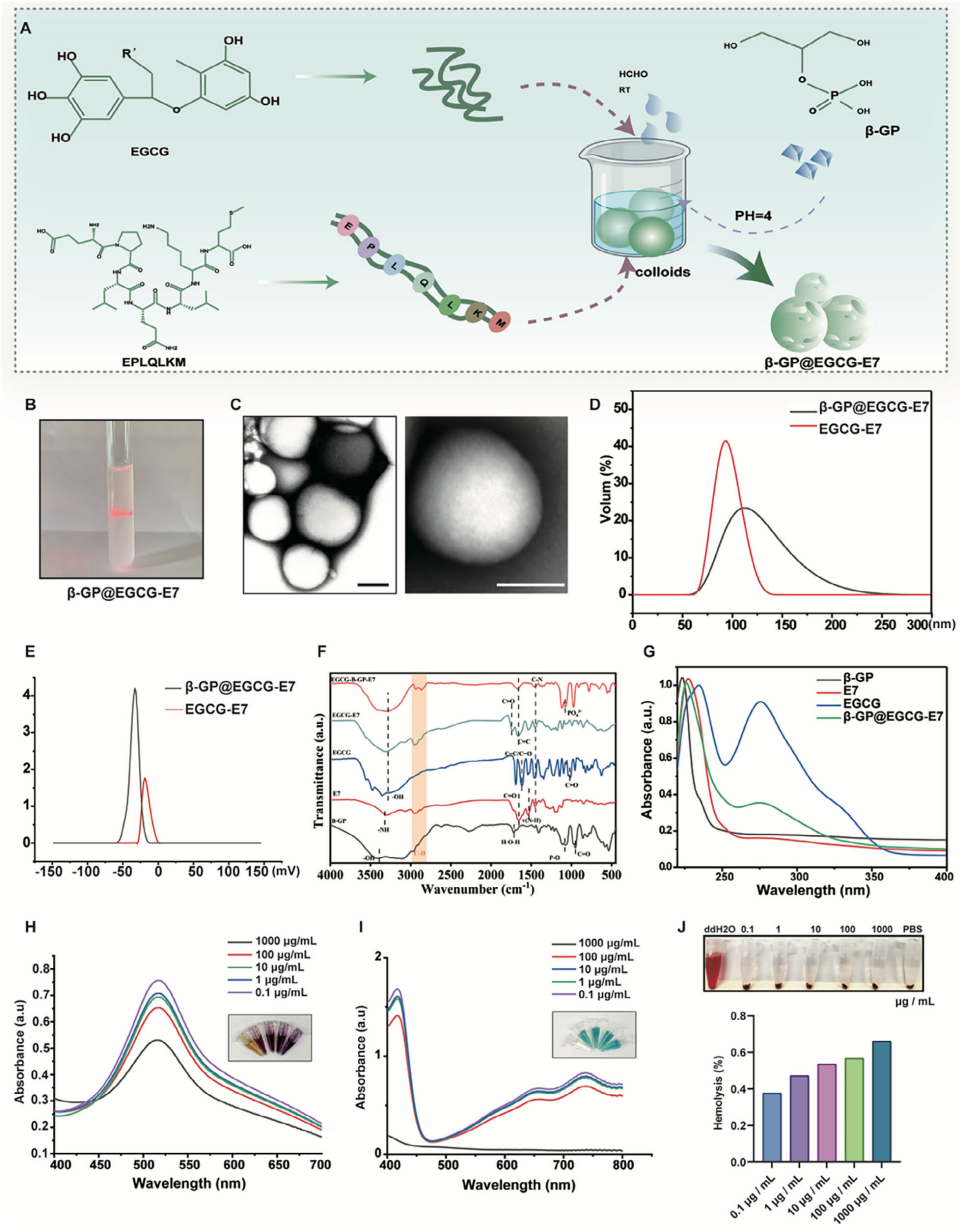
Characterization and functional evaluation of β‐GP@EGCG‐E7 nanoparticles. (A) Schematic illustration of the nanoparticle synthesis process. (B) Photograph of β‐GP@EGCG‐E7 solution exhibiting the Tyndall effect, confirming successful nanoparticle formation. (C) TEM image showing the morphology of β‐GP@EGCG‐E7 nanoparticles, scale bar = 100 nm. (D) Particle size distribution of β‐GP@EGCG‐E7 compared with EGCG‐E7, measured by dynamic light scattering. (E) Zeta potential analysis of β‐GP@EGCG‐E7 and EGCG‐E7 nanoparticles, illustrating surface charge changes upon β‐GP loading. (F) FTIR spectra of β‐GP, EGCG, EGCG‐E7, and E7, showing characteristic peaks and successful conjugation. (G) UV‐vis spectra further confirming successful integration of β‐GP, EGCG, and E7. (H,I) Antioxidant activity of β‐GP@EGCG‐E7 at different concentrations, evaluated by ABTS and DPPH assays. (J) Hemolysis assay and representative images of red blood cells treated with various concentrations of β‐GP@EGCG‐E7.

EGCG exhibits substantial reactivity and rapidly reacts with formaldehyde in an aqueous solution at room temperature, forming hydroxy‐methyl substituted EGCG [30]. Subsequently, amino or thiol groups undergo a Mannich condensation with the hydroxymethyl groups, resulting in the formation of EGCG colloidal spheres in the aqueous phase. In this study, EGCG was condensed with the E7 peptide in a formaldehyde solution, and the reaction mixture was stirred for 2 h, taking advantage of the free amino groups (the N‐terminal amine and the lysine side chain) in the E7 sequence, which serve as nucleophilic sites in the reaction. After the reaction, the resulting colloid was collected by centrifugation at 7000 rpm and washed three times with deionized water to remove unreacted components. Finally, sodium β‐glycerophosphate (β‐GP) was incorporated into the coating through a disassembly–reassembly process performed at pH 4.0 to generate the β‐GP@EGCG‐E7 nanodrug (Figure 1A). The resulting nanodrug displayed a distinct Tyndall effect, indicating successful formation of stable colloidal nanoparticles, consistent with previous reports on EGCG‐based self‐assembled nanostructures [30, 31] (Figure 1B). Transmission electron microscopy (TEM) revealed that the β‐GP@EGCG‐E7 nanoparticles possessed a uniform spherical morphology (Figure 1C). Dynamic light scattering (DLS) analysis showed that the hydrodynamic diameter increased from ∼90 to ∼110 nm following E7 condensation and β‐GP loading, whereas the zeta potential increased from ‐30 to −17 mV, implying surface modification and partial charge shielding rather than particle aggregation (Figure 1D,E). FTIR analysis confirmed that all three components were present in the same nanocomposite and interacted with one another. A broad O─H/N─H band at ∼3400 cm^−^^1^ was retained in β‐GP@EGCG‐E7, indicating hydrogen‐bonding networks. The C─H stretching band at ∼2900 cm^−^^1^ was intensified, consistent with β‐GP incorporation. The EGCG band near 1600 cm^−^^1^ overlapped with and was slightly blue‐shifted relative to the amide I/II bands of E7, indicating an altered microenvironment rather than a simple physical mixture. In addition, a new P–O (PO_4_^3^^−^) peak at ∼1000 cm^−^^1^ appeared exclusively in the composite, confirming the successful β‐GP integration [32]. Collectively, these findings agree with the dynamic light scattering and zeta‐potential results and support the formation of the β‐GP@EGCG‐E7 nanocomposite (Figure 1F). UV–vis spectroscopy further verified the coexistence of multiple components (Figure 1G). EGCG exhibited a characteristic aromatic polyphenol band at 270–280 nm, whereas E7 displayed peptide‐associated absorption at 210–230 nm. The β‐GP@EGCG–E7 nanocomposite retained both regions and presented a broadened band around 280 nm, implying that EGCG remained in a slightly altered electronic environment after assembly. Because β‐GP has minimal absorption above 250 nm, these signals primarily originated from EGCG and E7, indicating that β‐GP did not disrupt but instead supported EGCG‐E7 co‐assembly (Figure 1G). Release studies further showed that the nanocomposite displayed component‐dependent release behavior. EGCG was released more rapidly under physiological pH 7.4 than under acidic conditions, reaching a cumulative release of nearly 60% within 72 h, whereas its release at pH 5 remained lower and plateaued at around 30%. By comparison, the E7 peptide exhibited a more gradual release pattern, with cumulative release increasing over time under both conditions and showing slightly higher release at neutral pH than at acidic pH over 72 h (Figure S1A). In parallel, phosphate release from β‐GP was strongly influenced by ALP activity: higher ALP concentrations markedly accelerated phosphate generation, whereas only minimal release was observed in the absence of enzyme, supporting an ALP‐responsive mineralization process rather than simple passive leakage (Figure S1B). The physicochemical stability of β‐GP@EGCG‐E7 was also evaluated under different conditions. The particle size remained relatively stable at pH 7.4 over 72 h, whereas more obvious enlargement was observed under acidic conditions (pH 5.0), suggesting that the nanostructure was more stable in a physiological environment but became more dynamic under acidic conditions (Figure S1C). Similarly, in serum‐free Hank's Balanced Salt Solution (HBSS), the hydrodynamic size changed only slightly over time, while a more pronounced increase was detected in HBSS containing 10% FBS, likely reflecting interactions with serum proteins and formation of a protein corona rather than rapid structural collapse (Figure S1D). These results indicate that the nanocomposite maintains acceptable colloidal stability under biologically relevant conditions. The antioxidant assays further demonstrated that the radical‐scavenging activity of EGCG was largely preserved after nano‐assembly. The DPPH solution changed from deep purple to yellow, and the ABTS solution transitioned from blue to gray in the presence of β‐GP@EGCG‐E7 (Figure 1H,I). The nanodrug also exhibited a hemolysis rate below 5% at all tested concentrations and showed no significant cytotoxicity toward MC3T3‐E1 or RAW 264.7 cells, as confirmed by live‐dead staining and CCK‐8 assays after 5 days. These results demonstrate excellent biocompatibility and biosafety for further biomedical evaluation (Figure 1J; Figure S1E–H). Overall, this study successfully prepared the β‐GP@EGCG‐E7 nanodrug, which possesses a well‐defined spherical structure, high colloidal stability, robust antioxidant activity, and excellent biocompatibility, providing a strong material foundation for its potential use in bone‐related regeneration and modulation of inflammatory microenvironments.

### 2.2 β‐GP@EGCG‐E7 Exhibits Anti‐Inflammatory and Antioxidant Effects In Vitro

**FIGURE 2:**
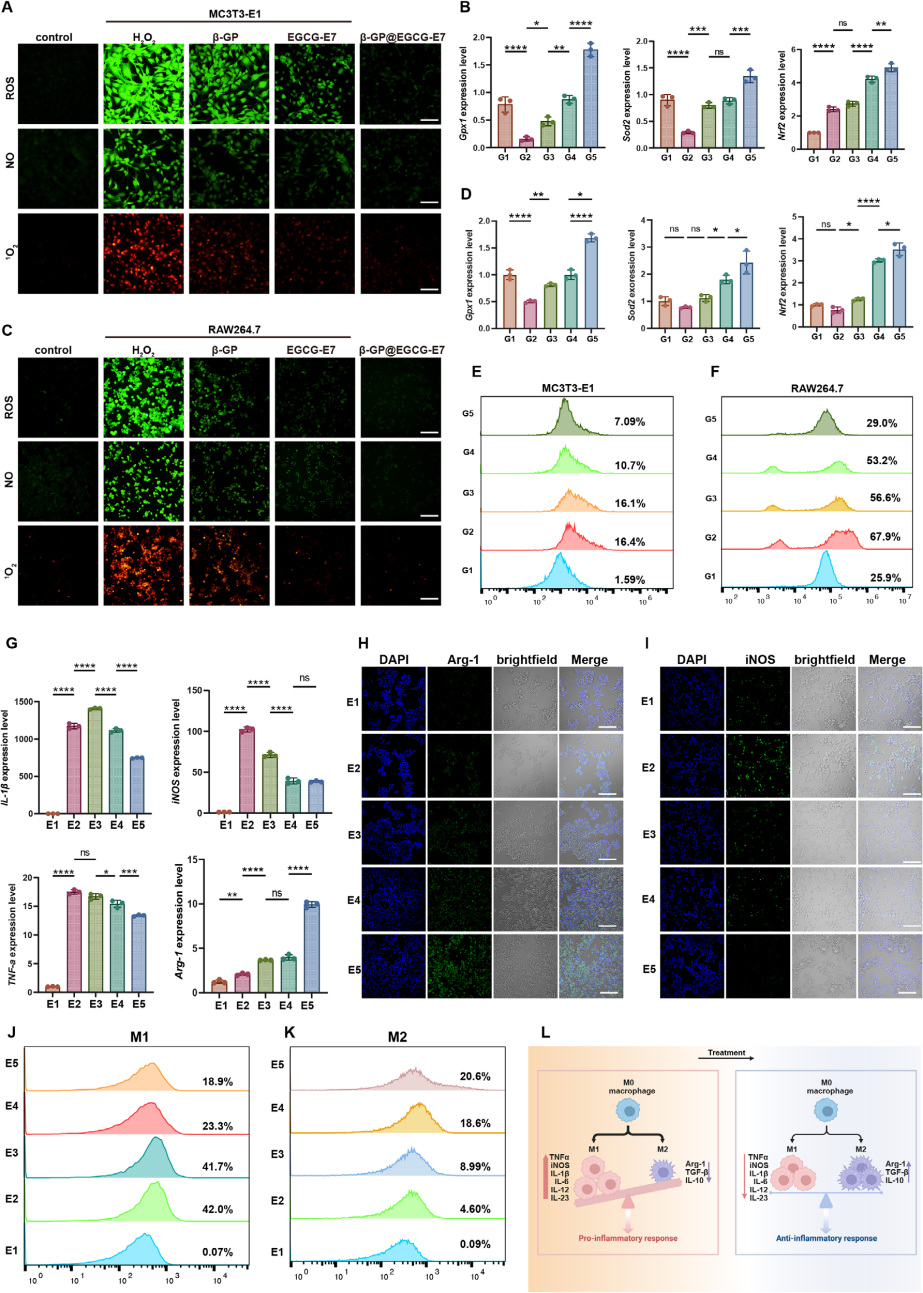
Antioxidant and anti‐inflammatory effects of β‐GP@EGCG‐E7 in MC3T3‐E1 and RAW264.7 cells. (A,C) Representative fluorescence images showing intracellular ROS, ^1O_2_, and NO in MC3T3–E1 (A) and RAW264.7 (C) cells after H_2_O_2_ challenge and treatment with different formulations. Scale bar = 200 µm. (B, D) qPCR analysis of antioxidant‐related gene expression (*Sod2, Nrf2*, and *Gpx1*) in MC3T3‐E1 (B) and RAW264.7 (D) cells. Data are presented as mean ± standard deviation (*n* = 3); Statistical significance was determined by one‐way ANOVA followed by Tukey's post hoc test (^*^ *p* < 0.05, ^**^ *p* < 0.01, ^***^ *p* < 0.001, and ^****^ *p* < 0.0001, ns = not significant). G1–G5 represent the Control, H_2_O_2_, β‐GP, EGCG‐E7, and β‐GP@EGCG‐E7 groups, respectively. (E,F) Flow cytometric quantification of intracellular ROS levels in MC3T3–E1 (E) and RAW264.7 (F) cells. (G) qPCR analysis of inflammatory gene expression in RAW264.7 cells, including pro‐inflammatory markers (*TNF‐α, IL‐1β*, and *iNOS*) and the anti‐inflammatory marker *Arg‐1*. Data are presented as mean ± standard deviation (*n* = 3); Statistical analysis was performed using one‐way ANOVA followed by Tukey's post hoc test (^*^ *p* < 0.05, ^***^ *p* < 0.001, ^****^ *p* < 0.0001, ns = not significant). (H,I) Immunofluorescence staining of iNOS and Arg‐1 in RAW264.7 cells. Scale bar = 200 µm. G1–G5 represent the Control, LPS, β‐GP, EGCG‐E7, and β‐GP@EGCG‐E7 groups, respectively. (J, K) Flow cytometric analysis of M1/M2 macrophage polarization following LPS (M1) or IL‐4 (M2) stimulation, with or without nanoparticle treatment. (L) Schematic diagram illustrating the modulation of macrophage polarization from M1 to M2 by β‐GP@EGCG‐E7.

Within the hyperglycemic, pro‐inflammatory milieu of diabetes, sustained oxidative stress and inflammation play central roles in disease progression [33, 34]. To evaluate the antioxidant effects of β‐GP@EGCG‐E7, EGCG‐E7, and β‐GP, we first induced oxidative stress by treating MC3T3‐E1 and RAW264.7 cells with 100 µm H_2_O_2_ for 2 h. A probe panel covering distinct reactive species was selected, including DCFH‐DA for total ROS, DAF‐FM DA for NO, and SOSG for singlet oxygen (^1^O_2_), thereby avoiding reliance on a single oxidant measurement and better reflecting the mixed oxidative burden characteristic of diabetic tissues. As expected, H_2_O_2_ markedly increased the generation of ROS, NO, and ^1^O_2_ in both cell types. Compared with the H_2_O_2_ group, EGCG‐E7 and β‐GP@EGCG‐E7 both reduced oxidative fluorescence signals, whereas β‐GP alone showed only limited effects. Among the tested formulations, β‐GP@EGCG‐E7 consistently produced the greatest reduction in ROS‐, NO‐, and ^1^O_2_‐related fluorescence in both MC3T3‐E1 and RAW264.7 cells (Figure 2A,C). Subsequent qPCR analysis further demonstrated that β‐GP@EGCG‐E7, most effectively upregulated expression of the core regulatory gene *Nrf2* and its downstream effectors *Gpx1* and *Sod2* in both MC3T3‐E1 and RAW264.7 cells, whereas β‐GP alone induced only modest changes (Figure 2B,D). These genes collectively enhance peroxide detoxification and superoxide elimination, thereby strengthening the endogenous antioxidant defense system [33, 34, 35, 36]. Flow cytometry corroborated these findings, showing that β‐GP@EGCG‐E7 reduced the ROS‐positive population from 16.4% to 7.09% in MC3T3‐E1 cells and from 67.9% to 29.0% in RAW264.7 cells, whereas EGCG‐E7 showed a weaker but still detectable effect (10.7% and 53.2%, respectively) and β‐GP alone produced little improvement (16.1% and 56.6%) (Figure 2E,F). Together, these results indicate that β‐GP@EGCG‐E7 exerts the strongest antioxidant activity among the tested groups. In addition, diabetic conditions favor an M1‐dominant inflammatory state, generating a persistent cytokine milieu that impairs cellular recruitment, suppresses endothelial neovascularization, and hinders bone repair [37]. To assess anti‐inflammatory activity, we used a RAW264.7 macrophage model stimulated with 1 µg/mL LPS. Successful establishment of this model was confirmed by a marked increase in iNOS and a decrease in Arg‐1 expression, as shown by immunofluorescence staining. Treatment with EGCG‐E7 and especially β‐GP@EGCG‐E7 reversed these changes by decreasing iNOS and increasing Arg‐1, whereas β‐GP alone showed a much weaker effect (Figure 2H,I). qPCR analysis further validated these findings, showing that β‐GP@EGCG‐E7 markedly downregulated the pro‐inflammatory genes *IL‐1β, TNF‐α*, and *iNOS*, while upregulating the anti‐inflammatory gene *Arg‐1*, providing transcriptional evidence of its potent inflammation‐resolving activity (Figure 2G). This shift in gene expression, consistent with a transition from an M1‐dominant to an M2‐favoring phenotype, was directly confirmed by macrophage polarization assays. β‐GP@EGCG‐E7 showed the most pronounced regulatory effect, reducing the M1 population from 42.0% to 18.9% and increasing the M2 population from 4.60% to 20.6%, while EGCG‐E7 produced an intermediate effect (M1, 23.3%; M2, 18.6%) (Figure 2J,K). Taken together, in an in vitro hyperglycemia‐ and inflammation‐mimicking diabetic model, β‐GP@EGCG‐E7 showed the strongest antioxidant and anti‐inflammatory activity, outperforming both β‐GP and EGCG‐E7 (Figure 2L).

### 2.3 β‐GP@EGCG‐E7 Promotes Osteogenesis and Angiogenesis Under Hyperglycemic and Inflammatory Conditions

**FIGURE 3:**
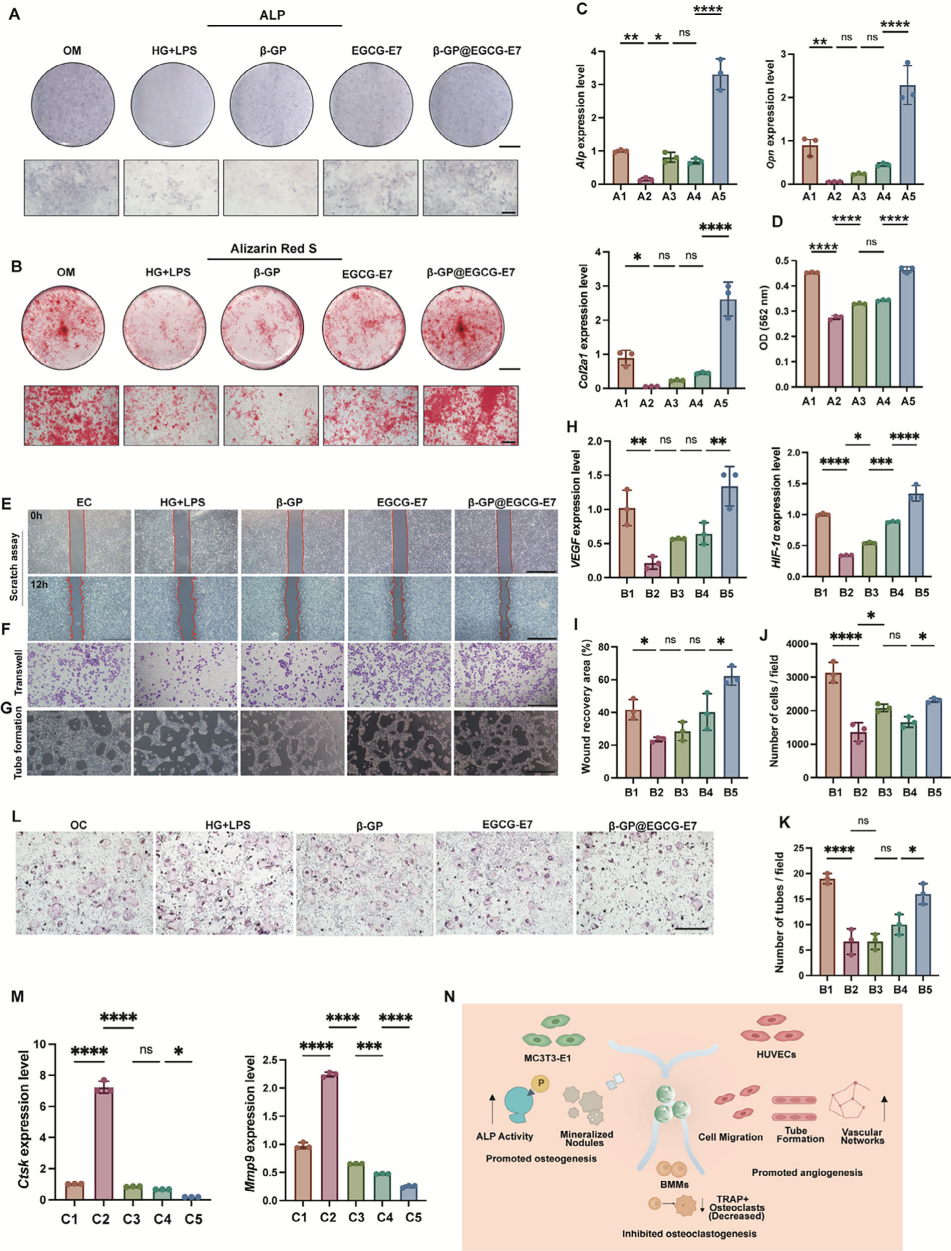
Effects of β‐GP@EGCG‐E7 on osteogenic differentiation and angiogenesis. (A) Representative ALP staining of MC3T3–E1 cells under different conditions: osteogenic medium (OM), high‐glucose/high‐inflammatory (OM+HG+LPS) group, OM+β‐GP, OM+EGCG‐E7, and OM+β‐GP@EGCG‐E7. Scale bar = 3 mm (upper panel), 200 µm (lower panel). (B) Alizarin red S (ARS) staining of MC3T3–E1 cells across the five groups. Scale bar = 3 mm (upper panel), 200 µm (lower panel). (C) qPCR analysis of osteogenic gene expression (*Col2a1, Opn*, and *Alp*) among groups. Data are presented as mean ± standard deviation (*n* = 3); Statistical significance was assessed by one‐way ANOVA followed by Tukey's post hoc test (^*^ *p* < 0.05, ^***^ *p* < 0.001, ^****^ *p* < 0.0001, ns = not significant). A1–A5 represent the OM, OM+HG+LPS, OM+β‐GP, OM+EGCG‐E7, and OM+β‐GP@EGCG‐E7 groups, respectively. (D) Semi‐quantitative analysis of ARS staining intensity across groups (one‐way ANOVA, ^****^ *p* < 0.0001, ns = not significant). A1–A5 represent the OM, OM+HG+LPS, OM+HG+LPS+β‐GP, OM+HG+LPS+EGCG‐E7, and OM+HG+LPS+β‐GP@EGCG‐E7 groups, respectively. (E) Representative bright‐field images from the scratch‐wound‐healing assay showing differences in cell migration. Scale bar = 500 µm. (F) Bright‐field images of the transwell migration assay. Scale bar = 500 µm. (G) Representative images of tube formation by HUVECs under different treatments. Scale bar = 500 µm. (H) qPCR analysis of angiogenesis‐related genes (*Notch1* and *VEGF*). Data are presented as mean ± standard deviation (*n* = 3); Statistical significance was assessed by one‐way ANOVA followed by Tukey's post hoc test (^*^ *p* < 0.05, ^***^ *p* < 0.001, ^****^ *p* < 0.0001, ns = not significant). E1–E5 represent the endothelial cell medium (ECM), ECM +HG+LPS, ECM +β‐GP, ECM +EGCG‐E7, and ECM +β‐GP@EGCG‐E7 groups, respectively. (I) Quantitative analysis of wound closure under hyperglycemic inflammatory conditions. Data are presented as mean ± standard deviation (*n* = 3); Statistical significance was assessed by one‐way ANOVA followed by Tukey's post hoc test (^*^ *p* < 0.05, ns = not significant). (J) Quantitative analysis of endothelial cell migration. Statistical significance was assessed by one‐way ANOVA followed by Tukey's post hoc test (^*^ *p* < 0.05, ^****^ *p* < 0.0001, ns = not significant). (K) Quantitative analysis of tube formation under hyperglycemic inflammatory conditions. Data are presented as mean ± standard deviation (*n* = 3); Statistical significance was assessed by one‐way ANOVA followed by Tukey's post hoc test (^*^ *p* < 0.05, ^****^ *p* < 0.0001, ns = not significant). (L) Representative images of the osteoclast induction under different treatments. Scale bar = 500 µm. (M) Quantitative analysis of osteoclast induction under different treatments. Data are presented as mean ± standard deviation (*n* = 3); Statistical significance was assessed by one‐way ANOVA followed by Tukey's post hoc test (^*^ *p* < 0.05, ^****^ *p* < 0.0001, ns = not significant). C1–C5 represent the osteoclast induction medium (OCM), OCM +HG+LPS, OCM +β‐GP, OCM +EGCG‐E7, and OCM +β‐GP@EGCG‐E7 groups, respectively. (N) Schematic illustration of the mechanism by which β‐GP@EGCG‐E7 alleviates hyperglycemic inflammation and promotes osteogenesis and angiogenesis.

Given that oxidative stress and chronic inflammation are central drivers of impaired bone healing in diabetes [35, 36], and that β‐GP@EGCG‐E7 has already been shown to attenuate excessive inflammation and reduce peroxide‐induced injury, we hypothesized that β‐GP@EGCG–E7 nanoparticles could restore osteogenesis and angiogenesis through coordinated regulation of the diabetic bone microenvironment. To investigate their osteogenic regulatory effects under hyperglycemic conditions, we established an in vitro diabetic surrogate by supplementing basal medium with 35 mmol/L D‐glucose and 1 µg/mL LPS, thereby recapitulating a hyperglycemic, pro‐inflammatory milieu. MC3T3‐E1 cells were then treated with β‐GP, EGCG‐E7, and β‐GP@EGCG‐E7 and subjected to osteogenic induction for 7 and 21 days. The hyperglycemic and pro‐inflammatory environment markedly suppressed osteogenic activity, as evidenced by reduced ALP secretion and minimal calcium nodule formation. Conversely, β‐GP@EGCG‐E7 treatment resulted in dense ALP‐positive deposits and abundant deep‐red mineralized nodules, whereas the β‐GP and EGCG‐E7 groups showed only partial recovery (Figure 3A,B). Quantitative analysis further confirmed these results, showing that alizarin red S (ARS) absorbance was significantly higher in the β‐GP@EGCG‐E7 group than in the other groups (Figure 3D). Consistently, qPCR demonstrated that β‐GP@EGCG‐E7 significantly upregulated osteogenesis‐related genes, including *Alp, Opn*, and *Col2a1* indicating restored and enhanced osteogenic activity under hyperglycemic inflammation (Figure 3C). These findings reveal that the therapeutic potential of β‐GP@EGCG‐E7 extends beyond protecting osteoblasts from hyperglycemic injury to actively promoting their bone‐forming capacity in a high‐glucose environment. Since diabetes is frequently accompanied by microvascular dysfunction and impaired skeletal perfusion [40], we next evaluated the effects of hyperglycemic inflammation on endothelial behavior and the capacity of β‐GP@EGCG‐E7 to reverse these impairments. In the scratch‐wound assay, endothelial migration was markedly impaired under hyperglycemic conditions, resulting in a significantly larger residual wound area at 12 h. Conversely, β‐GP@EGCG‐E7 treatment markedly accelerated wound closure, with a significantly increased wound recovery area compared with the HG+LPS group; EGCG‐E7 also improved migration, whereas β‐GP showed a weaker effect (Figure 3E,I). In the transwell migration assay, the HG+LPS group showed a clear reduction in migrated cell number, while β‐GP@EGCG‐E7 significantly restored endothelial migration; β‐GP and EGCG‐E7 produced only partial improvement (Figure 3F,J). In the tube‐formation assay, endothelial cells cultured under hyperglycemic conditions formed sparse and discontinuous tubular networks, whereas β‐GP@EGCG‐E7 treatment promoted the formation of continuous, well‐organized capillary‐like structures with significantly increased tube numbers compared with the HG+LPS group (Figure 3G,K). These observations indicate that β‐GP@EGCG‐E7 effectively mitigates hyperglycemia‐induced endothelial dysfunction, accelerates the repair process, and enhances both endothelial migration and angiogenic capacity. Consistently, qPCR analysis revealed marked upregulation of *VEGF* and *HIF‐1α*, confirming enhanced angiogenic responsiveness (Figure 3H). Since diabetic bone loss also involves excessive osteoclast activity, we further examined whether β‐GP@EGCG‐E7 could regulate osteoclastogenesis. TRAP staining showed that hyperglycemic inflammatory conditions promoted osteoclast formation, whereas β‐GP@EGCG‐E7 markedly reduced the number of TRAP‐positive multinucleated osteoclasts; β‐GP and EGCG‐E7 showed weaker inhibitory effects (Figure 3L). qPCR analysis further demonstrated that β‐GP@EGCG‐E7 significantly downregulated the osteoclast‐related genes *Ctsk* and *Mmp9* (Figure 3M). Taken together, these results indicate that β‐GP@EGCG‐E7 restores osteoblast and endothelial function while suppressing osteoclastogenesis under diabetic conditions, thereby promoting bone–vascular coupling and rebalancing bone remodeling (Figure 3N).

### 2.4 β‐GP@EGCG‐E7 Attenuates Bone Loss and Vascular Dysfunction in DOP Mice

**FIGURE 4:**
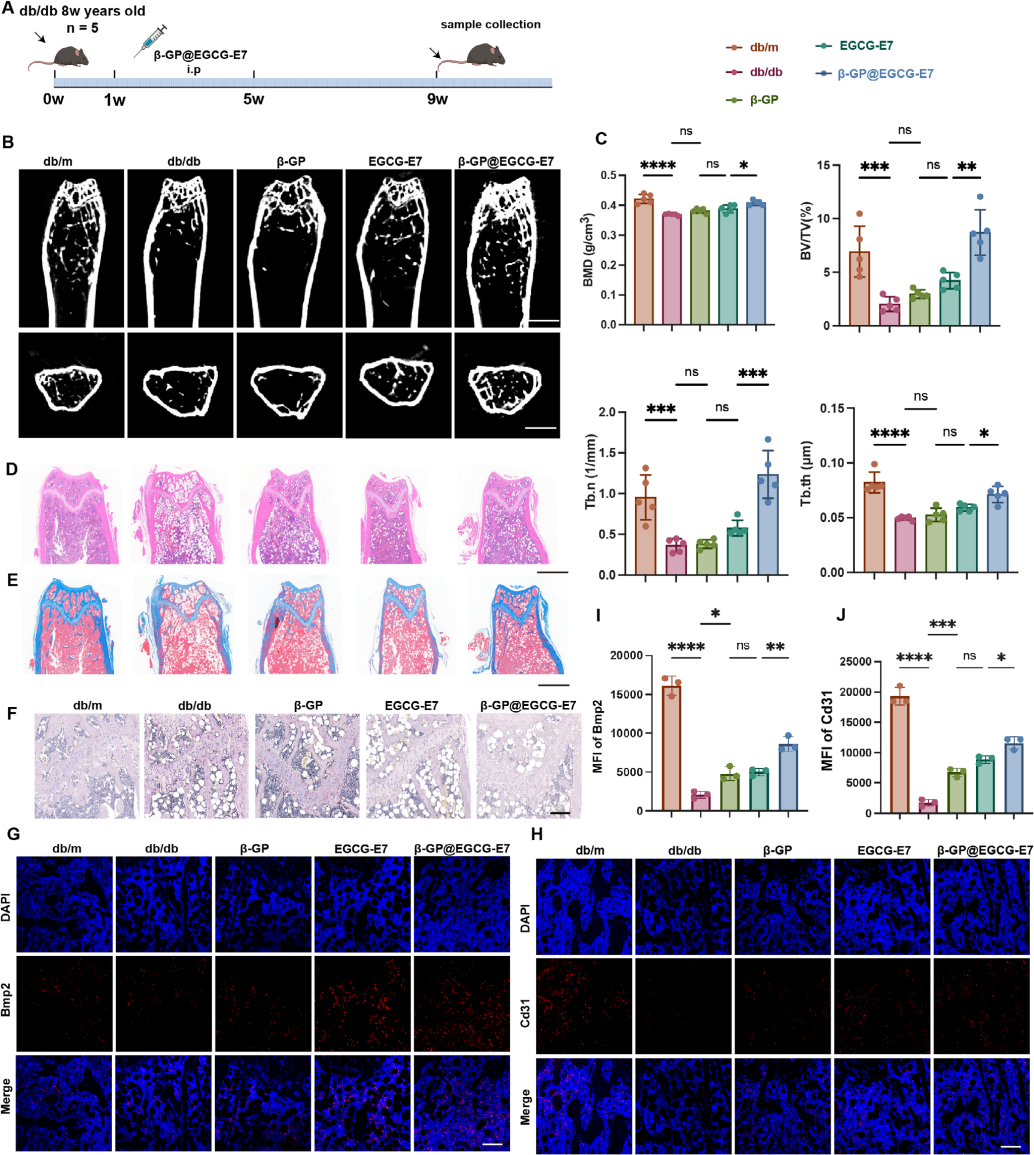
β‐GP@EGCG‐E7 alleviates bone loss and promotes osteogenic and angiogenic responses in db/db mice. (A) Experimental scheme for β‐GP@EGCG‐E7 treatment in db/db mice. (B) Representative micro‐CT images of femurs from db/m, db/db, β‐GP, EGCG‐E7, and β‐GP@EGCG‐E7 groups. (C) Quantitative analysis of bone mineral density (BMD), bone volume fraction (BV/TV), trabecular number (Tb.N), and trabecular thickness (Tb.Th). Data are presented as mean ± standard deviation (*n* = 5); Statistical significance was determined by one‐way ANOVA followed by Tukey's post hoc test (^*^ *p* < 0.05, ^**^ *p* < 0.01, ^***^ *p* < 0.001, and ^****^ *p* < 0.0001, ns = not significant). (D–F) Representative H&E, Masson's trichrome, and TRAP staining of femoral sections. D, E: Scale bar = 1 mm. F: Scale bar = 200 µm. (G, H) Representative immunofluorescence staining of BMP2 and CD31 in femoral sections. Scale bar = 200 µm. (J,K) Quantitative analysis of Bmp2 and Cd31 fluorescence intensity. Data are presented as mean ± SD (*n* = 3). Statistical significance was analyzed by one‐way ANOVA followed by Tukey's post hoc test. (^*^ *p* < 0.05, ^**^ *p* < 0.01, ^***^ *p* < 0.001, ^****^ *p* < 0.0001; ns, not significant).

Although the precise pathogenesis of DOP has not been fully elucidated, endocrine dysregulation together with persistent hyperglycemia and diabetes‐associated calcium loss are widely regarded as key drivers of diabetes‐related bone fragility [37, 38]. To evaluate whether β‐GP@EGCG‐E7 can ameliorate these diabetes‐induced skeletal impairments, we subsequently conducted comprehensive in vivo studies to rigorously assess its efficacy in alleviating bone loss and to elucidate its underlying anti‐inflammatory and antioxidant mechanisms. To better evaluate the therapeutic effect of β‐GP@EGCG‐E7 in a type 2 diabetes‐related osteoporosis, db/db mice were used for in vivo evaluation, with db/m mice serving as nondiabetic controls. β‐GP@EGCG‐E7 was administered by intraperitoneal injection, and femoral samples were collected at the end of treatment for micro‐computed tomography (micro‐CT), histological, and immunofluorescence analyses (Figure 4A). Longitudinal monitoring further showed that β‐GP@EGCG‐E7 did not markedly reduce body weight or blood glucose in db/db mice, suggesting that its skeletal benefit was unlikely to be mainly attributable to systemic glycemic correction (Figure S2A,B). Micro‐CT analysis revealed pronounced bone mass reductions and marked trabecular deterioration in the db/db group compared with the control group, confirming successful model establishment. Both β‐GP and EGCG‐E7 treatments showed a trend toward increased bone mass relative to the db/db group. Notably, β‐GP@EGCG‐E7 produced a significant increase in BMD, and key trabecular parameters, including bone volume fraction (BV/TV), trabecular number (Tb.N), and trabecular thickness (Tb.Th), were markedly improved. These findings indicate that β‐GP@EGCG‐E7 exerts a pronounced therapeutic effect in reversing diabetic bone deterioration and promoting bone regeneration (Figure 4B,C). Histological analyses corroborated the imaging results. H&E and Masson's trichrome staining showed improved trabecular integrity and enhanced collagen deposition in the β‐GP@EGCG‐E7‐treated group compared with db/db mice (Figure 4D,E). Notably, TRAP staining demonstrated a clear reduction in osteoclast‐positive cells, suggesting that β‐GP@EGCG‐E7 ameliorated diabetic bone loss not only by promoting bone formation, but also by suppressing osteoclastogenesis and bone resorption (Figure 4F). To investigate the mechanisms underlying β‐GP@EGCG‐E7‐mediated bone regeneration under diabetic conditions, immunofluorescence staining was performed for osteogenic and angiogenic markers. The osteogenic marker Bmp2 was markedly upregulated, and the angiogenic marker Cd31 was significantly increased in the β‐GP@EGCG‐E7 group, as confirmed by both representative staining and quantitative analysis (Figure 4G–J). Furthermore, immunofluorescence staining of inflammation‐ and oxidative stress‐related markers revealed marked alterations in the diabetic bone microenvironment. Compared with the db/db group, β‐GP@EGCG‐E7 treatment markedly increased the fluorescence intensities of Arg‐1, Nrf2, and Sod2, while reducing iNOS expression, as further confirmed by quantitative mean fluorescence intensity (MFI) analysis. Although β‐GP and EGCG‐E7 alone also partially modulated these markers, the combined β‐GP@EGCG‐E7 treatment showed the most pronounced effect, suggesting that it effectively remodeled the diabetic microenvironment and enhanced antioxidant defense in vivo (Figure S2C–J). Compared with the db/db group, β‐GP@EGCG‐E7 produced denser and more uniform positive staining, indicating effective promotion of osteogenic differentiation and angiogenesis within the hyperglycemic inflammatory microenvironment, thereby enhancing bone tissue regeneration and repair. To determine whether the in vivo efficacy of β‐GP@EGCG‐E7 extended beyond the db/db model, we further validated the nanoplatform in an independent STZ‐induced diabetic osteoporosis model. Notably, β‐GP@EGCG‐E7 produced a similar protective phenotype in STZ‐treated mice, as evidenced by restored bone mass, improved trabecular microarchitecture, enhanced osteogenic and angiogenic signaling within the femur, and reduced inflammation and oxidative stress in the bone microenvironment (Figure S3 and S4). Together with the results in db/db mice, these findings support the robustness of β‐GP@EGCG‐E7 across distinct diabetic bone osteoporosis.

### 2.5 β‐GP@EGCG‐E7 Restores Mitochondrial Quality Control by Reactivating PINK1‐Parkin–Mediated Mitophagy

**FIGURE 5:**
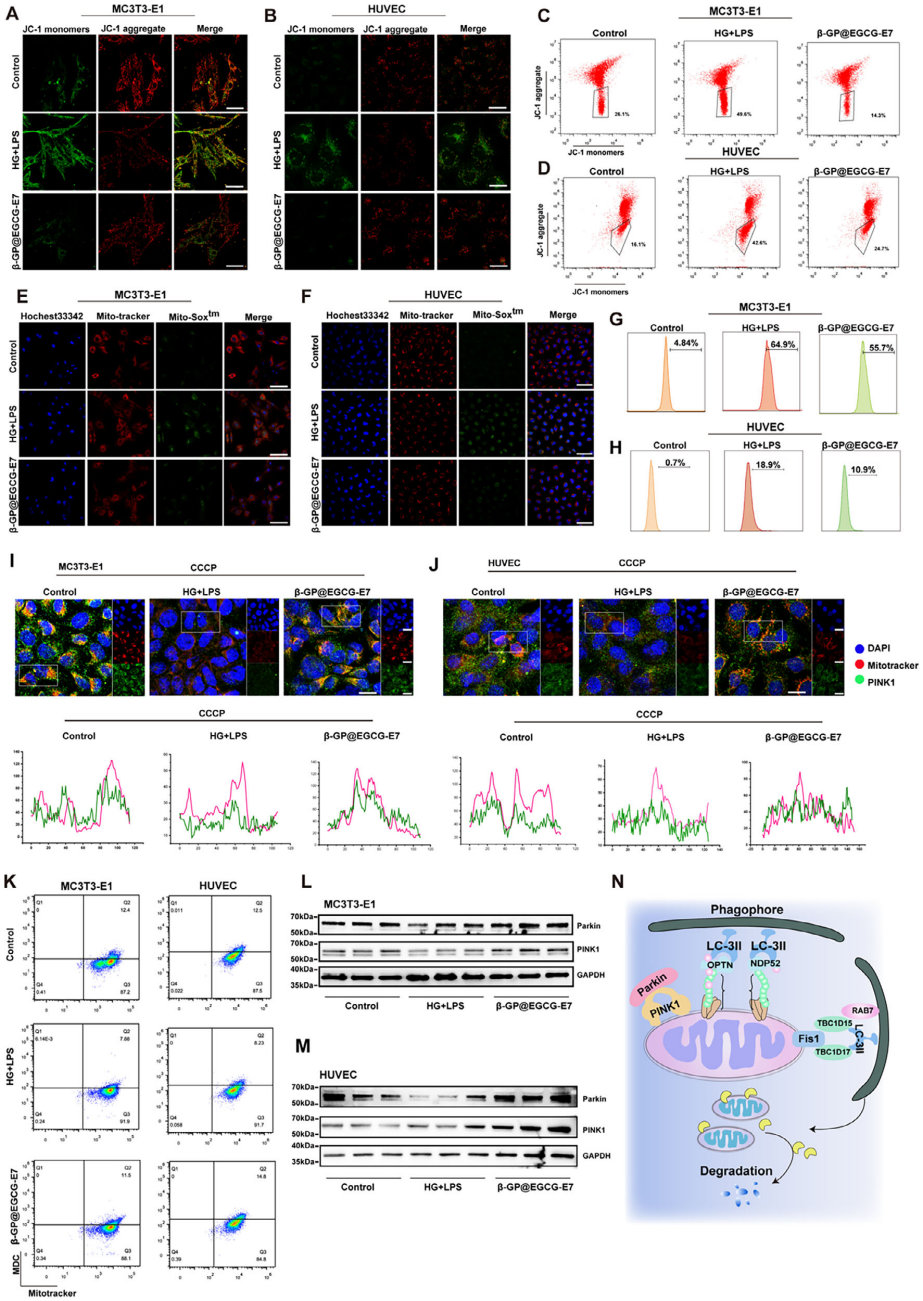
Regulation of mitochondrial function and mitophagy by β‐GP@EGCG‐E7 under high‐glucose/high‐inflammatory conditions. (A) Representative JC‐1 fluorescence images of MC3T3‐E1 cells in control, high‐glucose/high‐inflammatory, and β‐GP@EGCG‐E7 treatment groups, showing changes in mitochondrial membrane potential. Scale bar = 20 µm. (B) JC‐1 fluorescence images of HUVEC cells under the same three conditions. Scale bar = 20 µm. (C) Flow cytometric analysis of JC‐1 in MC3T3‐E1 cells across the three groups. (D) Flow cytometric analysis of JC‐1 in HUVEC cells across the three groups. (E,G) MitoSOX staining and flow cytometric quantification of mitochondrial ROS levels in MC3T3–E1 cells under the three conditions. Scale bar = 40 µm. (F,H) MitoSOX staining and flow cytometric quantification of mitochondrial ROS levels in HUVEC cells under the three conditions. Scale bar = 40 µm. (I) Representative immunofluorescence images showing colocalization of PINK1 (green) and MitoTracker (red) in MC3T3–E1 cells (scale bar = 20 µm, upper panel). Quantitative analysis of PINK1‐MitoTracker colocalization among control, HG + LPS, and β‐GP@EGCG‐E7 groups, expressed as fluorescence overlap coefficients (lower panel). (J) Representative immunofluorescence images showing colocalization of PINK1 (green) and MitoTracker (red) in HUVEC cells (scale bar = 20 µm, upper panel). Quantitative analysis of PINK1‐MitoTracker colocalization among control, HG + LPS, and β‐GP@EGCG‐E7 groups (lower panel). (K) Autophagy assessment by MDC staining and MitoTracker double labeling under the three conditions using flow cytometry. (L,M) Western blot analysis of autophagy‐related markers PINK1 and Parkin in MC3T3–E1 and HUVEC cells. (N) Schematic illustration of the mechanism by which β‐GP@EGCG‐E7 restores mitophagy under high‐glucose and pro‐inflammatory conditions.

The pronounced osteogenic and angiogenic effects observed in diabetic animals prompted us to investigate further the mechanisms underlying the protective actions of β‐GP@EGCG‐E7. In diabetes, persistent hyperglycemia and inflammation disrupt cellular metabolic balance, induce excessive oxidative stress, and ultimately lead to mitochondrial dysfunction [39]. In turn, dysfunctional mitochondria become a major source of ROS, further amplifying oxidative damage and establishing a vicious cycle of mitochondrial injury, and impairment of mitochondrial quality control, particularly mitophagy, has been increasingly implicated in diabetes‐related tissue injury [40, 41, 42]. Therefore, restoring mitochondrial homeostasis and mitophagic function is considered a key mechanism for mitigating diabetes‐related cellular and tissue injury. To clarify the cytoprotective mechanism of β‐GP@EGCG‐E7 under hyperglycemic and inflammatory stress, we first assessed mitochondrial membrane potential using the JC‐1 assay. In MC3T3‐E1 cells and HUVECs exposed to high glucose and LPS, the red/green fluorescence ratio markedly decreased, reflecting mitochondrial depolarization and ΔΨm collapse. Treatment with β‐GP@EGCG‐E7 largely reversed this pattern, with an obvious reduction in green monomer fluorescence and a corresponding increase in red J‐aggregate fluorescence, indicating restoration of mitochondrial membrane potential and function (Figure 5A,B). Consistently, flow cytometric analysis further confirmed a significant decrease in the proportion of green‐positive (low ΔΨm) cells compared with the HG + LPS group, from 49.6% to 14.3% in MC3T3‐E1 cells and from 42.6% to 24.7% in HUVECs (Figure 5C,D). Because loss of ΔΨm is typically accompanied by excessive mitochondrial ROS production, we next examined mitochondrial oxidative stress. Mitochondrial ROS staining revealed that β‐GP@EGCG‐E7 treatment markedly reduced mitochondrial ROS accumulation, indicating a key role in mitigating oxidative stress (Figure 5E,F). Flow cytometry analysis further corroborated this finding, with mitochondrial ROS levels in MC3T3‐E1 cells decreasing from 64.9% to 55.7%, and in HUVEC, decreasing from 18.9% to 10.9% following treatment (Figure 5G,H). These findings imply that β‐GP@EGCG–E7 stabilizes mitochondrial function and attenuates oxidative‐stress under hyperglycemic and inflammatory conditions. Given that mitophagy is essential for mitochondrial quality control, we next examined whether the mitochondrial protective effects of β‐GP@EGCG‐E7 were associated with activation of mitophagy‐related pathways. Flow cytometric analysis showed that β‐GP@EGCG‐E7 increased the proportion of MDC/MitoTracker double‐positive cells in both MC3T3‐E1 cells and HUVECs compared with the HG + LPS group, supporting enhanced autophagy‐related activity (Figure 5K). Immunofluorescence co‐localization analysis further revealed enhanced overlap between PINK1 and Mitotracker signals in the β‐GP@EGCG‐E7‐treated group after CCCP (carbonyl cyanide 3‐chlorophenylhydrazone) induction under high‐glucose and high‐inflammatory conditions, indicating increased recruitment of PINK1 to damaged mitochondria and activation of the mitophagic cascade (Figure 5I,J). As PINK1 accumulation on dysfunctional mitochondria promotes Parkin translocation, we further evaluated the PINK1‐Parkin pathway. Western blot analysis demonstrated a recovery of PINK1 and Parkin expression following β‐GP@EGCG‐E7 treatment under high‐glucose and inflammatory conditions (Figure 5L,M). To further test whether this pathway was functionally involved in the pro‐regenerative effects of β‐GP@EGCG‐E7, we introduced chloroquine (CQ) to inhibit autophagy. Under CQ treatment, the promotive effects of β‐GP@EGCG‐E7 on osteogenesis and angiogenesis were markedly attenuated, as reflected by reduced expression of osteogenic markers and angiogenesis‐related genes, impaired wound healing and migration, and weakened mineralization capacity (Figure S5A–E). In parallel, CQ also diminished the β‐GP@EGCG‐E7‐induced recovery of PINK1 and Parkin expression (Figure S5F). Collectively, β‐GP@EGCG‐E7 protects osteoblasts and endothelial cells from hyperglycemia‐ and inflammation‐induced injury by re‐establishing mitochondrial homeostasis and promoting PINK1‐Parkin‐mediated mitophagy. This mechanism not only preserves mitochondrial function but also alleviates oxidative stress, providing a promising therapeutic approach for diabetic osteoporosis. These findings suggest that the mitochondrial protective and pro‐regenerative effects of β‐GP@EGCG‐E7 are at least partly dependent on mitophagy‐related signaling, providing mechanistic support for its therapeutic potential in diabetic osteoporosis (Figure 5N).

### 2.6 Biocompatibility and Biodistribution of β‐GP@EGCG–E7

**FIGURE 6:**
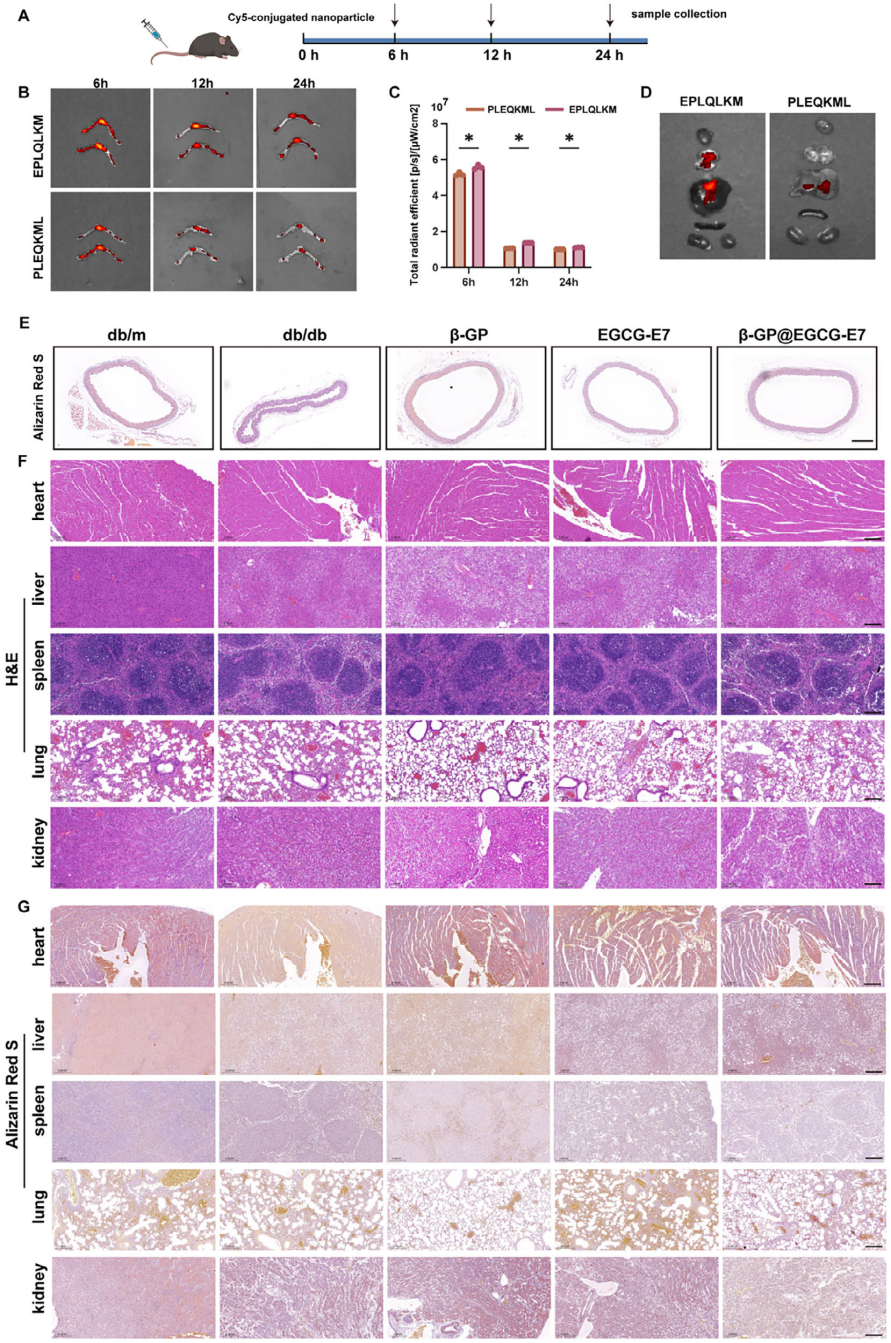
Biodistribution specificity and biosafety evaluation of β‐GP@EGCG‐E7 in vivo. (A) Schematic illustration of Cy5‐labeled nanoparticle administration and tissue collection for ex vivo fluorescence imaging. (B) Representative ex vivo fluorescence images of harvested bone samples collected at 6, 12, and 24 h after administration of nanoparticles modified with the E7 sequence (EPLQLKM) or the scrambled peptide sequence (PLEQKML). (C) ROI‐based quantitative analysis of fluorescence intensity in harvested bone tissues at different time points. Data are presented as mean ± standard deviation (*n* = 3); Statistical significance was determined by one‐way ANOVA followed by Tukey's post hoc test (^*^ *p* < 0.05). (D) Representative ex vivo fluorescence images of collected tissues from the E7 and scrambled‐peptide groups. (E) Alizarin Red S staining of arterial sections. Scale bar = 200 µm. (F) H&E staining of major organs, including the heart, liver, spleen, lung, and kidney. Scale bar = 200 µm. (G) Alizarin Red S staining of major organs for evaluation of ectopic calcification. Scale bar = 200 µm.

Following extensive in vitro and in vivo evaluations demonstrating the therapeutic potential of β‐GP@EGCG‐E7, we further assessed its biosafety and biodistribution through comprehensive biological safety analyses and ex vivo fluorescence imaging (Figure 6A). To further determine whether bone accumulation was associated with the targeting capability of E7 rather than nonspecific adsorption, Cy5‐labeled nanoparticles modified with the E7 sequence (EPLQLKM) were compared with nanoparticles carrying a scrambled peptide sequence (PLEQKML). ex vivo imaging of harvested skeletal tissues showed that the E7‐modified formulation exhibited consistently stronger fluorescence signals than the scrambled control at 6, 12, and 24 h after systemic administration (Figure 6B). In addition, supplementary comparison between free β‐GP and β‐GP@EGCG‐E7 also showed stronger skeletal accumulation of the nanoplatform than β‐GP alone, further supporting the bone‐targeting advantage conferred by nanoassembly (Figure S6A–C). Quantitative analysis further confirmed significantly higher bone‐associated radiant efficiency in the E7 group over time (Figure 6C), supporting the contribution of E7 to skeletal targeting. After 24 h, most β‐GP@EGCG‐E7 had undergone metabolic clearance, with only minimal residual signal in the liver and lungs, indicating that β‐GP@EGCG‐E7 exhibits favorable in vivo elimination and does not accumulate excessively in off‐target organs (Figure 6D). This clearance profile reduces the likelihood of long‐term toxicity and supports its suitability for repeated or long‐term administration. We next evaluated the biosafety of repeated systemic administration in db/db mice. Alizarin Red S staining of arterial sections did not reveal obvious ectopic calcium deposition in any treatment group (Figure 6E). H&E staining of the heart, liver, spleen, lung, and kidney showed preserved tissue architecture without evident inflammation, necrosis, or pathological injury after β‐GP@EGCG‐E7 treatment (Figure 6F). In addition, Alizarin Red S staining of these major organs did not detect pathological calcification in non‐bone tissues (Figure 6G). To verify the biosafety of drug injection in STZ‐induced diabetic mice, we further performed H&E staining of major organs, which likewise showed no obvious histopathological damage after treatment (Figure S6D). Collectively, these results indicate that β‐GP@EGCG‐E7 displays favorable in vivo biosafety together with enhanced bone‐associated accumulation, supporting its further development for diabetic osteoporosis therapy.

## 3 Discussion

In this study, we constructed a multifunctional colloidal nanoplatform by condensing the bone‐targeting peptide E7 with EGCG using a polypeptide‐polyphenol conjugation strategy, followed by loading the resulting assembly with the osteogenic agent β‐GP. Compared with conventional amino acid‐polyphenol systems, this platform showed markedly enhanced structural stability and functional integration. More importantly, unlike many previously reported DOP‐directed biomaterials or nanomedicines that mainly intervene along a single therapeutic axis, β‐GP@EGCG‐E7 was designed as an integrated self‐assembled system that coordinates bone targeting, redox buffering, inflammatory microenvironment modulation, angiogenic support, and phosphate‐assisted osteogenesis within one platform. It provided bone‐specific targeting, antioxidant capacity, and phosphate‐mediated osteogenic stimulation within a single system. Under the pathological conditions of DOP, the nanoplatform not only alleviated oxidative stress and restored osteoblast function but also repaired vascular endothelial injury, improved local microcirculation, and restored mitochondrial quality control and mitophagy through activation of the PINK1‐Parkin pathway, thereby promoting bone repair and regeneration. Notably, the newly added CQ inhibition experiments further support that these beneficial effects are at least partly dependent on mitophagy activity, thereby strengthening the mechanistic link between mitochondrial quality control and the observed pro‐regenerative phenotype. Recent work has shown that Parkin acetylation‐mediated mitophagy can orchestrate periodontal ligament stem cell osteogenesis and bone regeneration during ageing, further supporting the importance of mitophagy regulation in skeletal repair [43]. Another recent study reported that calcium silicate promotes BMSC osteogenesis and bone regeneration by inducing mitophagy‐mediated metabolic reprogramming toward oxidative phosphorylation, which is consistent with our observation that restoration of mitochondrial quality control is closely associated with improved osteogenic outcomes [44]. However, since the current evidence is based on pharmacological inhibition rather than direct genetic intervention, the precise contribution of PINK1–Parkin signaling to the therapeutic actions of β‐GP@EGCG‐E7 still warrants further validation. In addition, our supplementary osteoclast‐related findings suggest that β‐GP@EGCG‐E7 not only restores osteoblast and endothelial cell function, but also partially suppresses osteoclast differentiation and bone resorption, indicating that its therapeutic benefit may arise from a broader rebalancing of diabetic bone remodeling rather than from a purely anabolic effect alone. These combined effects may contribute to improved bone repair and regeneration in DOP. Importantly, the therapeutic efficacy of this platform was observed not only in the STZ‐induced diabetic osteoporosis model, but also in db/db mice, suggesting that its beneficial effects may extend beyond a hyperglycemia‐driven injury setting to a metabolically more complex type 2 diabetes context characterized by insulin resistance and chronic systemic dysregulation.

Despite these encouraging findings, several translational limitations should be acknowledged. First, although the efficacy of β‐GP@EGCG‐E7 was supported in both STZ‐induced and db/db mouse models, these murine systems still do not fully recapitulate the clinical heterogeneity of human type 2 diabetes–related osteoporosis, which is shaped by longer disease duration, aging, obesity, diverse comorbidities, and more complex microvascular complications. Moreover, the STZ model primarily reflects insulin‐deficient diabetic bone injury, whereas many patients with DOP present with insulin resistance, obesity, and broader metabolic dysregulation; thus, future validation in aged, diet‐induced, or other clinically more relevant models will be important for defining the real translational scope of this strategy. Although our current data did not reveal obvious ectopic calcification or major histopathological injury under the tested regimen, this should be regarded as short‐ to mid‐term safety reassurance rather than definitive evidence of long‐term safety, especially given that β‐GP is a mineralization precursor and repeated exposure may still carry tissue‐specific risks not captured within the present observation period. In addition, while the assembled nanoplatform showed acceptable colloidal stability under biologically relevant conditions, the time‐dependent chemical stability of EGCG itself and the retention of antioxidant activity in complex biological environments remain incompletely defined. This issue is particularly relevant for translation because it may influence storage robustness, batch consistency, and pharmacological reproducibility. Finally, although the current β‐GP exposure falls within a preliminarily manageable translational range, definitive clinical dosing, safety margins, pharmacokinetics, and dose‐escalation parameters remain to be established before systemic administration of β‐GP@EGCG‐E7 can be realistically advanced toward clinical application.

## 4 Materials and Methods

### 4.1 β‐GP@EGCG‐E7 Preparation

#### 4.1.1 Synthesis of EGCG Colloids

A total of 10 mg of EGCG (Solarbio, China) and 20 mg of the E7 peptide (Sangon Biotech, China) were dissolved in 10 mL of deionized water and stirred at 600 rpm until fully dissolved. Subsequently, 200 µL of 10% formaldehyde (Aladdin, China) was added dropwise, and the mixture was stirred at 600 rpm for 2 h at room temperature. The resulting colloidal particles were collected by centrifugation at 7000 g for 10 min and washed three times with deionized water. The purified colloidal particles were stored at 4°C for later use.

#### 4.1.2 Nanodrug Preparation

The colloidal particles were redispersed in deionized water, and β‐GP (Sigma–Aldrich, USA) was added to the colloidal suspension with thorough mixing. The pH of the mixture was adjusted to 4.0 using HCl to induce self‐assembly of the nanodrug. For example, to prepare β‐GP@EGCG‐E7 nanodrug, 100 µL of colloidal solution (40 mg/mL) was mixed with 100 µL of β‐GP aqueous solution (40 mg/mL). After thorough mixing, β‐GP@EGCG‐E7 nanodrug was obtained at a final concentration of 20 mg/mL.

### 4.2 Material Characterization

FT‐IR spectra were recorded on a Thermo Nicolet iS20 spectrometer using the KBr pellet method, with a scan range of 4000–500 cm^−^^1^. TEM images were acquired using a JEM‐1400 transmission electron microscope. UV–vis spectra were measured on a Thermo Scientific SPECTRONIC 200 ultraviolet–visible spectrophotometer, and characteristic absorption peaks were used to confirm molecular interactions and successful conjugation. Particle size, polydispersity index, and zeta potential were determined using a ZETASIZER NANO ZSP at a refractive index of 1.52 and a temperature of 25°C. Each measurement lasted 120 s and was averaged over 30 cycles.

### 4.3 DPPH Scavenging Assay

In a 1‐mL reaction system, 100 µL of a 1 mm DPPH ethanol solution (Solarbio, BC4750) was mixed with sample solutions at various concentrations. The mixture was incubated at room temperature in the dark for 30 min, followed by centrifugation. The absorbance of the supernatant was measured at 519 nm to evaluate free radical scavenging activity.

### 4.4 ABTS Scavenging Assay

The assay was conducted according to the instructions of the Total Antioxidant Capacity Assay Kit (Beyotime, S0119). The absorbance of the final reaction solution was measured at 734 nm to determine ABTS radical scavenging activity.

### 4.5 Cell Viability Assay

RAW264.7 and MC3T3–E1 cells were cultured in DMEM supplemented with 10% FBS and 1% penicillin–streptomycin at 37°C in a humidified atmosphere containing 5% CO_2_, with passaging every 3 days. Cells were seeded in 96‐well plates at a density of 2000 cells per well and incubated overnight to allow attachment. Various concentrations of β‐GP@EGCG‐E7 were then added to each well. After incubation for 24, 72, or 120 h, 10 µL of CCK‐8 solution (Beyotime, C0037) was added to each well and incubated for an additional 45 min. Absorbance was measured at 450 nm to determine cell viability.

### 4.6 Live/Dead Staining

MC3T3–E1 and RAW264.7 cells were seeded in 24‐well plates at a density of 5 × 10^4^ cells per well. After cell attachment, the culture medium was replaced with basal medium containing the specified concentrations of nanomaterials, and the cells were incubated for 24 h. The cells were then washed three times with PBS and stained with Calcein/PI working solution (Beyotime, C2015M) following the manufacturer's instructions. After incubation at 37°C for 30 min, fluorescence images were captured using an inverted fluorescence microscope (Olympus IX73).

### 4.7 Hemolysis Assay

Fresh blood was collected from C57BL/6J mice and centrifuged at 1000 rpm for 5 min to isolate red blood cells (RBCs). The RBCs were washed three times with PBS and resuspended in PBS to prepare a 2% hematocrit suspension. Various concentrations of nanoparticles were incubated with the RBC suspension at 37°C for 4 h. Following incubation, hemolysis was terminated by placing the samples on ice, and the suspension was centrifuged at 1000 rpm for 5 min. Hemoglobin release in the supernatant was quantified by measuring absorbance at 540 nm using a microplate reader.

### 4.8 ALP and ARS Staining

To assess osteogenic differentiation and mineralization in MC3T3–E1 cells, alkaline ALP and ARS staining were performed. For ALP staining, cells were seeded in 24‐well plates at a density of 2 × 10^5^ cells per well and cultured in complete medium containing the different materials for 7 days, followed by staining according to the manufacturer's instructions (Beyotime, P0321S). For ARS staining, cells were cultured in complete medium with the different materials for 14 days, with the medium refreshed every 3 days. After fixation, cells were stained with ARS solution (Solarbio, G8550) to evaluate calcium deposition. The bound dye was then solubilized using 1% dimethylpyridine, and absorbance was measured at 450 nm for quantitative analysis.

### 4.9 Scratch Wound Healing Assay

HUVECs were seeded in six‐well plates at a density of 5 × 10^5^ cells per well. Once a confluent monolayer had formed, a sterile 100‐µL pipette tip was used to create a straight scratch across the cell layer. Detached cells were removed by washing three times with PBS, and the cultures were maintained in serum‐free medium containing the test materials. Wound closure was imaged at 0 and 24 h using an Olympus IX73 inverted microscope, and the percentage of wound closure over time was calculated to evaluate cell migration.

### 4.10 Tube Formation Assay

Each well of a 48‐well plate was coated with 120 µL of Matrigel and allowed to solidify. HUVECs were then seeded onto the Matrigel at a density of 1 × 10^5^ cells per well and treated with the indicated experimental conditions. The plates were incubated at 37°C in a 5% CO_2_ atmosphere for 6 h to allow the formation of capillary‐like structures. Tube formation was subsequently assessed and imaged using an optical microscope (Olympus IX73).

### 4.11 Transwell Migration Assay

HUVECs were seeded in the upper chamber of an 8‐µm‐pore‐size Transwell insert (Biofil, China) at a density of 2 × 10^5^ cells per well. The upper chamber contained serum‐free DMEM supplemented with the indicated materials, whereas 500 µL of complete medium was added to the lower chamber to serve as a chemoattractant. After 12 h of incubation, non‐migrated cells on the upper surface of the membrane were gently removed with a sterile swab. Migrated cells on the underside of the membrane were fixed with 4% paraformaldehyde, stained with 0.1% crystal violet for 10 min, and visualized under a light microscope (Olympus IX73). Representative images were captured for subsequent quantitative analysis.

### 4.12 Preparation of Bone Marrow‐Derived Cells and Osteoclastogenesis Assay

Male C57BL/6J mice aged 6–8 weeks were euthanized, and their femurs and tibias were aseptically harvested. After removal of attached soft tissue, the bone marrow was flushed from the medullary cavity with ice‐cold α‐MEM containing 1% penicillin/streptomycin. The obtained suspension was passed through a 70 µm cell strainer to remove tissue fragments and generate a single‐cell preparation. Cells were pelleted by centrifugation at 300 × g for 5 min at 4°C, followed by red blood cell lysis with Solarbio lysis buffer (R1010). After washing, the recovered bone marrow cells were used for osteoclast induction. Tartrate‐resistant acid phosphatase staining and related induction procedures were performed with a commercial osteoclast assay kit (Amizona, AMK1001‐24T, Hangzhou Yangming Biotechnology Co., Ltd.) following the supplier's protocol.

### 4.13 Immunofluorescence Staining of Mouse Femoral Tissues

Frozen femur sections from mice were sequentially fixed, permeabilized, and blocked prior to immunostaining. Sections were incubated overnight at 4°C with primary antibodies, including ALP (1:200, Abcam), CD31 (1:200, Invitrogen), iNOS (1:200, Proteintech), Arg1 (1:200, Proteintech), Nrf2 (1:200, Abclonal), and Gpx1 (1:200, Abclonal). Following three washes with PBS, sections were incubated with Alexa Fluor 488‐ or 594‐conjugated secondary antibodies (1:500, Invitrogen) for 1 h at room temperature. Nuclei were counterstained with DAPI (Beyotime, C1005). Fluorescence images were acquired using an Olympus FV3000 confocal microscope under consistent imaging parameters.

### 4.14 Immunofluorescence Staining of MC3T3–E1 Cells and HUVECs

MC3T3–E1 and HUVEC cells were cultured with the indicated materials for 24 h and subsequently treated with CCCP (MCE, HY‐100941) for 2 h to induce mitochondrial damage. Cells were then fixed, permeabilized, and blocked, followed by incubation with a primary antibody against PINK1 (1:500, Proteintech) overnight at 4°C. After washing, cells were incubated with Alexa Fluor 488‐conjugated secondary antibodies (1:500, Invitrogen) for 1 h at room temperature, and nuclei were counterstained with DAPI (Beyotime, C1005). Fluorescence imaging was performed using an Olympus FV3000 confocal microscope under standardized settings.

### 4.15 Immunofluorescence Staining of RAW264.7 Cells

RAW264.7 cells were stimulated with LPS (MCE, HY‐D1056) to induce an inflammatory phenotype and subsequently co‐cultured with the indicated materials. Following treatment, cells were fixed, permeabilized, and blocked, and then incubated with primary antibodies against iNOS (1:200, Abcam) and Arg1 (1:200, Proteintech) overnight at 4°C. After washing, cells were incubated with Alexa Fluor 488‐conjugated secondary antibodies (1:500, Invitrogen) for 1 h at room temperature, and nuclei were counterstained with DAPI (Beyotime, C1005). Fluorescence images were acquired using an Olympus FV3000 confocal microscope under consistent imaging parameters.

### 4.16 Measurement of Intracellular ROS, NO, ^1O2, and Mitochondrial Function

Intracellular levels of ROS (Beyotime, S0035S), nitric oxide (NO) (Beyotime, S0021S), singlet oxygen (^1O_2_) (Beyotime, S0060), mitochondrial membrane potential (ΔΨm) (Beyotime, C2006), and mitochondrial superoxide (Beyotime, S0061) were measured and quantified according to the manufacturers’ protocols using the respective assay kits.

### 4.17 QPCR and Western Blot

Total RNA was extracted using Trizol reagent (Sigma–Aldrich, T9424). Reverse transcription was performed using a PrimeScript RT Kit (Takara, RR037A), followed by quantitative PCR according to the manufacturer's instructions. Primer sequences are provided in Table S1.

Cells were lysed, and protein concentrations were determined using a BCA Protein Assay Kit (Beyotime, P0012). Western blot analysis was performed following standard protocols, including SDS‐PAGE, transfer onto PVDF membranes, blocking, and sequential incubation with primary and secondary antibodies. The primary antibodies used were PINK1 (1:1000, Proteintech), Parkin (1:1000, Proteintech), and GAPDH (1:10 000, Affinity Biosciences). HRP‐conjugated secondary antibodies (1:5000, Affinity Biosciences) were applied, and protein bands were visualized using chemiluminescence reagents (Epizyme, SQ201) on a ChemiDoc Touch Imaging System (Bio‐Rad).

### 4.18 Animal Experiments and Surgery

Eight‐week‐old male C57BL/6J mice were acclimated for 1 week under specific‐pathogen‐free conditions at 24 ± 2°C with 55 ± 5% relative humidity. After a 12‐h fast, the mice received a single intraperitoneal injection of STZ (Solarbio, 18883‐66‐4) at 180 mg/kg, dissolved in 0.1 mM citrate buffer (pH 4.2). Following STZ administration, the animals were fed a high‐sugar, high‐fat diet. During this period, treatments were administered three times per week for 4 consecutive weeks. Eight‐week‐old male db/db mice, purchased from GemPharmatech Co., Ltd, were included as an additional type 2 diabetic mouse model.

### 4.19 Histological Analysis

All major tissues were fixed in 4% paraformaldehyde, cryoprotected using a sucrose gradient, and embedded in optimal cutting temperature compound. Frozen sections of 10 µm thickness were prepared and subsequently subjected to H&E staining (Solarbio, G11120) and Masson's trichrome staining (Solarbio, G1340) to assess tissue morphology and fibrosis. Images were captured using a 3DHISTECH digital slide scanner.

### 4.20 Micro‐CT Analysis

After sample preparation, mouse femurs were carefully dissected and fixed in 4% paraformaldehyde for 48 h. High‐resolution micro‐CT scanning (Bruker SkyScan 1276) was performed, followed by three‐dimensional reconstruction. Quantitative analyses were conducted to assess BMD, BV/TV, Tb.Th, Tb.Sp, trabecular pattern factor (Tb.Pf), and Tb.N.

### 4.21 Statistical Analysis

All experiments were independently repeated three times. Data are presented as mean ± standard deviation. Statistical analyses were performed using GraphPad Prism 9.0 (GraphPad Software, USA). Comparisons among multiple groups were conducted using one‐way ANOVA. Statistical significance was defined as p < 0.05, with thresholds indicated as follows: *p < 0.05, ^**^ *p* < 0.01, ^***^ *p* < 0.001, ^****^ *p* < 0.0001; ns, not significant.

### 4.22 Ethical Declarations

All animal experiments were performed in full compliance with relevant guidelines and regulations. The study protocols were approved by the Institutional Animal Care and Use Committee (IACUC) of Peking University Health Science Center (Approval No. DLASBE0459). All procedures were conducted in accordance with the ARRIVE guidelines.

## Author Contributions

X.Y.X. contributed to experiment design, execution, and interpretation. M.Q.Z., T.W., E.F.W., A.Y.H., and Z.Y.W. performed the experiments. Z.J.W. and L.T.L. contributed to sample preparation, data visualization, and figure editing. L.K.W., X.T.P., Q.Y.Z., and X.Y.D. assisted with data curation, statistical analysis, and manuscript revision. H.L. and Y.S.Z. designed the experiments and oversaw the collection and interpretation of results. All authors have read and approved the final version of the manuscript.

## Conflicts of Interest

The authors declare no conflicts of interest.

## Supporting information




**Supporting File**: advs75666‐sup‐0001‐SuppMat.docx.

## Data Availability

The data supporting the findings of this study can be obtained from the corresponding author upon making a reasonable request.
