# Supplementary material for: Orchestrated Engineered Polyphenol‐Peptide Condensates Coupled With β‐GP Reverses Diabetic Osteoporosis by Remodeling Mitophagy
Source: Adv Sci (Weinh). 2026 May 12;13(44):e75666. doi: 10.1002/advs.75666 (PMC13336025; doi:10.1002/advs.75666)
Supplement: Supplementary file 1 [file ADVS-13-e75666-s001.docx]

Orchestrated engineered polyphenol-peptide condensates coupled with β-GP reverses diabetic osteoporosis by remodeling mitophagy

Xiuyun Xu^1,3^, Meiqin Zhang^1,3^, Ting Wu^2,3^, Erfan Wei^1,3^, Andrei Y. Hancharou^4^, Zeying Wang^1,3^, Zijuan Wang^2,3^, Letian Lv^2,3^, Likun Wu^1,3^, Xingtong Pan^1,3^, Qiyue Zhu^1,3^, Xinyi Dong^1,3^, Hao Liu^2, 3*^, Yongsheng Zhou^1, 3*^

1. Department of Prosthodontics, Peking University School of Stomatology, Beijing, 100081, China.
2. The Central Laboratory, Peking University School and Hospital of Stomatology, Beijing 100081, China
3. National Center of Stomatology, National Clinical Research Center for Oral Diseases, National Engineering Research Center of Oral Biomaterials and Digital Medical Devices, Beijing Key Laboratory for Intelligent Biomanufacturing and Regeneration of Craniofacial Tissues, Beijing Key Laboratory of Digital Stomatology, Research Center of Engineering and Technology for Computerized Dentistry Ministry of Health, NMPA Key Laboratory for Dental Materials, Beijing 100081, China
4. Institute of Biophysics and Cell Engineering, National Academy of Sciences of Belarus, 27 Akademicheskaya Street, 220072 Minsk, Republic of Belarus.

*Correspondence: [kqzhouysh@hsc.pku.edu.cn](mailto:kqzhouysh@hsc.pku.edu.cn), kqliuhao@bjmu.edu.cn

Figure S1

**Figure S1**

(A) EGCG release was evaluated at pH 5.0 and pH 7.4, while E7 release was evaluated at pH 5.0 and pH 7.4.

(B) Phosphate (Pi) release from β-GP in the presence of different concentrations of alkaline phosphatase (ALP; 0, 0.2, and 2 U/mL), demonstrating ALP-responsive phosphate generation.

(C) Time-dependent changes in particle size of the nanocomposite at pH 5.0 and pH 7.4.

(D) Colloidal stability of the nanocomposite in HBSS with or without 10% FBS, assessed by hydrodynamic size over 72 h.

(E, F) Cell viability of MC3T3–E1 and RAW264.7 cells after incubation with various concentrations of β-GP@EGCG-E7 for 24, 72, and 120 h, measured using the CCK-8 assay.

(G, H) Representative fluorescence images of (C) MC3T3–E1 and (D) RAW264.7 cells following exposure to increasing concentrations of β-GP@EGCG-E7. Live cells were stained with calcein-AM (green), and dead cells were stained with propidium iodide (red). Scale bar = 200 µm.

Figure S2

Figure S2

(A, B) Changes in body weight and blood glucose during treatment.

(C–F) Representative immunofluorescence staining of inflammatory (iNOS, Arg-1) and antioxidant stress-related markers (Nrf2, Sod2) in femoral sections.

(G–J) Quantitative analysis of the corresponding fluorescence intensities. Data are presented as mean ± SD (n = 3). Statistical significance was assessed by one-way ANOVA followed by Tukey’s post hoc test (*p < 0.05, ***p < 0.001, ****p < 0.0001, ns = not significant). Scale bar = 200μm.

Figure S3

Figure S3

(A) Schematic illustration of DOP mouse model establishment.

(B) Representative 2D micro-CT images of femurs from different experimental groups. Scale bar = 1mm.

(C) Quantification of femoral bone parameters obtained from micro-CT analysis, including BMD, BV/TV, Tb.Th, and Tb.N. Statistical significance was determined by one-way ANOVA (*p < 0.05, **p < 0.01, ***p < 0.001, ****p < 0.0001; ns, not significant).

(D) Representative histological images of femoral sections stained with hematoxylin and eosin (H&E), Masson’s trichrome, and Safranin O/Fast Green, showing trabecular microarchitecture, collagen deposition, and cartilage matrix distribution across groups. Scale bar = 500μm.

(E, F) Immunofluorescence staining of ALP and CD31 in femoral sections, indicating osteogenic activity and neovascularization. Scale bar = 200 μm.

(G, H) Quantitative analysis of ALP and CD31 fluorescence intensity in bone sections. Statistical significance was determined by one-way ANOVA (*p < 0.05, **p < 0.01, ***p < 0.001, ****p < 0.0001; ns = not significant).

Figure S4

Figure S4

(A) Immunofluorescence staining of Arg1 and iNOS in femoral sections, demonstrating anti-inflammatory activity within bone tissue. Scale bar = 200 μm.

(B) Immunofluorescence staining of GPX1 and Nrf2 in femoral sections, illustrating antioxidant activity within bone tissue. Scale bar = 200 μm.

(C) Quantitative analysis of fluorescence area for Arg1 and iNOS in bone sections. Statistical significance was determined by one-way ANOVA (*p < 0.05, **p < 0.01, ***p < 0.001, ****p < 0.0001; ns = not significant).

(D) Quantitative analysis of fluorescence area for GPX1 and Nrf2 in bone sections. Statistical significance was determined by one-way ANOVA (*p < 0.05, **p < 0.01, ***p < 0.001, ****p < 0.0001; ns = not significant).

Figure S5

Figure S5

(A) Relative expression levels of the angiogenesis-related genes VEGF and HIF-α in the Control, HG+LPS, HG+LPS+NP, and HG+LPS+NP+CQ groups. Data are presented as mean ± SD. Statistical significance was determined by one-way ANOVA followed by Tukey’s post hoc test (****p < 0.00001).

(B) Representative images of the scratch wound healing assay at 0 and 12 h, showing cell migratory capacity in the Control, HG+LPS, HG+LPS+NP, and HG+LPS+NP+CQ groups. Scale bar =200 μm.

(C) Relative expression levels of the osteogenesis-related genes Col2a1 and Sp7 in the the Control, HG+LPS, HG+LPS+NP, and HG+LPS+NP+CQ groups. Data are presented as mean ± SD. Statistical significance was determined by one-way ANOVA followed by Tukey’s post hoc test (**p < 0.01, ****p < 0.0001).

(D) Representative ALP staining images showing early osteogenic differentiation under different treatments. Scale bar =200 μm.

(E) Representative Alizarin Red S staining images showing extracellular matrix mineralization under different treatments. Scale bar =200 μm.

(F) Western blot analysis of Parkin and PINK1 protein expression under different treatments, with GAPDH used as the loading control.

Figure S6

Figure S6

(A) Schematic illustration of the experimental design for assessing *ex vivo* biodistribution and biosafety of β-GP@EGCG-E7 nanoparticles.

(B) *Ex vivo* fluorescence imaging of femurs at 6, 12, and 24 h post-injection, showing nanoparticle accumulation over time.

(C) *Ex vivo* fluorescence imaging of major organs (heart, liver, spleen, lung, and kidneys) at 24 h post-injection, indicating clearance and tissue distribution.

(D) H&E staining of major organs from mice after one month of treatment, showing intact tissue structure with no evident histopathological abnormalities, demonstrating good systemic biocompatibility of β-GP@EGCG-E7. Scale bar = 500 μm.

**Table S1. Primers used for gene expression analysis**

| Gene | Primer name | Sequence (5′–3′) |
| --- | --- | --- |
| *Alp (mouse)* | Alp-F | CTTGACTGTGGTTACTGCTG |
|  | Alp-R | GAGCGTAATCTACCATGGAG |
| *Opn (mouse)* | Opn-F | TCAAGTCAGCTGGATGAACC |
|  | Opn-R | CTTGTCCTTGTGGCTGTGAA |
| *Col2a1* *(mouse)* | Col2a1-F | CATCCAGGGCTCCAATGATGTA |
|  | Col2a1-R | ATGTCCATGGGTGCGATGTC |
| *VEGF (human)* | VEGF-F | AGGGAAGAGGAGGAGATGAG |
|  | VEGF-R | GCTGGGTTTGTCGGTGTT |
| *HIF-1a (human)* | HIF-1α-F | ACGTTCCTTCGATCAGTTGTCACC |
|  | HIF-1α-R | GGCAGTGGTAGTGGTGGCATTAG |
| *GAPDH (human)* | GAPDH-F | CTTTGGTATCGTGGAAGGACTC |
|  | GAPDH-R | GTAGAGGCAGGGATGATGTTCT |
| *iNOS (mouse)* | iNOS -F | CCAAGCCCTCACCTACTTCC |
|  | iNOS -R | CTCTGAGGGCTGACACAAGG |
| *IL-1β (mouse)* | IL-1β-F | AGAGCATCCAGCTTCAAATCTC |
|  | IL-1β-R | CAGTTGTCTAATGGGAACGTCA- |
| *Arg-1 (mouse)* | Arg1-F | AGTGTGGTGCTGGGTGGAGAC |
|  | Arg1-R | GCTGGTTGTCAGGGGAGTGTTG |
| *Tnf-α (mouse)* | Tnf-α-F | CCTCTTCTCATTCCTGCTTGTGG |
|  | Tnf-α-R | GGCCATTTGGGAACTTCTCATC |
| *Gapdh (mouse)* | Gapdh-F | TGCACCACCAACTGCTTA GC |
|  | Gapdh-R | GGCATGGACTGTGGTCATGAG |
